# Lightweight, Heat-Insulating, Alkali-Activated Slag Composites with Carbon-Based Biochar Additive and Filler

**DOI:** 10.3390/ma19020277

**Published:** 2026-01-09

**Authors:** Gintautas Tamošaitis, Danutė Vaičiukynienė, Aras Kantautas, Ignacio Villalón Fornés, Ruben Paul Borg, Laura Vitola

**Affiliations:** 1 Faculty of Civil Engineering and Architecture, Kaunas University of Technology, Studentų St. 48, 51367 Kaunas, Lithuania; gintautas.tamosaitis@ktu.lt (G.T.); ignacio.villalon@ktu.lt (I.V.F.); 2 Faculty of Industrial Engineering and Technology, Lietuvos Inžinerijos Kolegija|Higher Education Institution, Tvirtovės al. 35, 50155 Kaunas, Lithuania; aras.kantautas@lik.tech; 3 Faculty for Built Environment, University of Malta, MSD 2080 Msida, Malta; ruben.p.borg@um.edu.mt; 4 Faculty of Civil and Mechanical Engineering, Riga Technical University, Kipsalas St. 6A, 1048 Riga, Latvia; laura.vitola_1@rtu.lv

**Keywords:** metallurgical slag, biochar waste, alkali-activated materials, acoustic insulation, thermal conductivity

## Abstract

**Highlights:**

**What are the main findings?**

- Biochar waste (BW) was made from waste wood.
- A small amount of BW (>0.5%) was used as an additive, while a larger amount (1–25%) was used as a filler.
- Samples with 0.25% of BW exhibited the highest compressive strength (44.4 MPa).

**What are the implications of the main finding?**

- Based on SEM findings, BW had good adhesion with the alkali-activated slag matrix.
- The thermal and acoustic insulation effect of the samples increased with the addition of BW.

**Abstract:**

An alkali-activated slag binder based on biochar was developed in this research. The biochar was produced from waste wood and is referred to as biochar waste (BW). In the alkali-activated slag system, a small amount of biochar (up to 0.5%) was used as an additive, and a larger amount (from 1% to 25%) was used as a filler. The influence of the biochar powder on compressive strength was determined. The hydrated samples were investigated using X-ray diffraction (XRD) analysis and scanning electron microscopy (SEM), and the thermal, acoustical properties, and hydration temperature were also determined. The compressive strength of the alkali-activated slag composite, especially after 7 days, was found to increase slightly due to the introduction of a small amount (0.05–0.5%) of BW powder. The powder in the alkali-activated slag matrix was distributed homogenously, resulting in a reduction in the crack propagation. A larger amount of BW led to a non-homogeneous distribution, and this resulted in a gradual reduction in compressive strength with increasing BW. The highest values of compressive strength at 28 days of hydration (44.4 MPa) were recorded for samples with 0.25% of BW. According to mathematical analysis methods, the compressive strength is mainly influenced by the specific surface area of the initial mix ingredients and the amount of BW additive. In the alkali-activated slag matrix, BW acted as an inert micro-filler, with the dilution effect possibly being the reason for the decrease in the hydration temperature. SEM analysis demonstrated that the BW had a good adhesion with the alkali-activated slag matrix. The thermal and acoustic insulation performance of samples with BW improved. These investigations suggest that BW can be successfully incorporated in alkali-activated material, resulting in low thermal conductivity and adequate acoustic insulation performance.

## 1. Introduction

Biochar is a type of charcoal that is produced from the pyrolysis of organic materials, such as wood, agricultural waste, or another biomass. During the pyrolysis process, the organic materials are heated in the absence of oxygen, leading to their breakdown into a mixture of volatile gases, liquid bio-oil, and a solid residue called biochar (BW). BW is composed mainly of carbon, along with some ash and other minerals. It has a high surface area and a porous structure that make it useful for a variety of applications, such as soil improvement, water filtration, and carbon sequestration.

Different applications of BW in cementitious materials are reported. Chen et al. [1] determined that the introducing of BW accelerated the hydration of ordinary Portland cement (OPC) due to its pozzolanic character and internal curing. The compressive strength increased with the addition of up to 2 wt.% BW and slightly decreased at higher amounts. In another study by Akhtar et al. [2], the addition of BW led to improvement in the mechanical properties of cement due to its filler effect, especially in the early hydration period. Dey et al. [3] reported that incorporating 0.5–2% biochar (by cement weight) significantly enhanced concrete durability. Compressive strength increased by up to 16%, water permeability decreased by 40%, thermal damage was mitigated, strength under elevated temperatures improved by 20%, and water tightness rose by 22–25%. Similar findings by Saccani et al. [4] confirm that incorporating small amounts of biochar (<2 wt%) improves compressive strength by about 15% after 28 days and reduces capillary water absorption, enhancing durability. In contrast, higher biochar contents decrease strength but increase dimensional stability and limit efflorescence.

As depicted by Gupta et al. [5], the fine BW particles act as a micro-filler and lead to the formation of hydration products. Barissov [6] discovered that, during the hydration process of concrete with BW additive, nucleation centres formed, which led to a densification of the concrete system. The influence of BW on the properties of cement composites was investigated by Maljaee et al. [7]. OPC mortar with BW addition had no drying shrinkage cracks and the filler accelerated hydration. The optimal amount of this filler was 2% of the OPC mass, and in this case, the mechanical properties improved. When the filler content was higher than 2%, the mechanical properties decreased significantly. Dixit et al. [8] investigated ultra-high-performance concrete with BW as a substitute of ordinary Portland cement (OPC). The BW powder improves hydration through internal hardening and nucleation. When 5% BW was incorporated in the mixture, the compressive strength (144 MPa) was relatively similar to the reference samples (150 MPa) after 28 days of hydration. The investigation of the microstructure showed a dense contact zone between the BW and the hardened cement paste. Regarding mortars, Yang and Wang [9] determined that OPC mortars with BW in the range of 5% had similar mechanical properties to the reference samples without BW at 28 days of hydration (only a 5.5% reduction in compressive strength). Moreover, BW exhibited a dilution effect and then a nucleation effect in the OPC systems.

Egodagamage et al. [10] determined that an alkali-activated slag system with rice husk BW led to an increase in compressive strength at the early and late hydration periods. The optimal amount of BW additive was found to be 2 wt.% and this additive improved the formation of hydration products. Han et al. [11,12] determined that the higher hydration degree of alkali-activated slag systems with BW is due to higher water capacity, cation exchange, and surface sorption. BW, as a porous material in alkaline-activated slag systems, first adsorbed water and then gradually released water during the hydration process. This process increased the mechanical properties and durability of the hydration alkali-activated slag system. Prabahar et al. [13] reported that the incorporation of BW (1.54% and 1.63% of slag mass) in alkali-activated slag led to an insignificant increase in compressive strength when using two types of alkali activators: NaOH and Na_2_CO_3_ solutions. Egodagamage et al. [10,14] investigated alkali-activated blends of ground granulated blast furnace slag and BW. A mixture of NaOH and Na_2_CO_3_ solutions was used as an alkaline activator. The optimum amount of rice husk BW was 2% and, in this case, a positive change in compressive strength was reported. The increase in compressive strength was explained through the specific internal curing of BW: high water absorption, retention, and release. A larger compressive strength (44.5%) was reported for the samples with BW when compared to concrete samples without BW. Piccolo et al. [15] investigated lightweight alkali-activated materials for which precursors were made from bottom ash, pure metakaolin, and large amounts of BW. It was determined that samples with 70 wt.% BW had higher total porosity with smaller pore sizes when compared with reference samples without BW. The authors attributed the decrease in pore size to the effect of BW filling on these pores. The authors also noticed that there was no slowdown of the geopolymerisation process. The sorption properties of geopolymers with BW (up to 30% weight) were investigated by Khamlue et al. [16]. Precursors of these geopolymers were made from metakaolin and aluminium oxide and a mixture of sodium silicate and sodium hydroxide solutions as alkali activators. The removal efficiency tests reached 60–70% and this corresponded to high adsorption capacity. Through this approach, it was possible to create additional sorption material for water purification.

Previous research has explored the use of biochar (BW) in construction materials primarily for sustainability purposes; however, its dual role as both an additive and a filler in alkali-activated systems remains insufficiently investigated. In this study, a special type of BW derived from waste wood was incorporated into alkali-activated slag mixtures. BW is an environmentally friendly material that not only enhances mechanical and durability properties but also contributes to the development of innovative, eco-friendly building insulation materials. This approach addresses the gap in the current literature and strengthens the connection between the introduction and subsequent sections of the article.

## 2. Materials and Methods

### 2.1. Experimental Techniques

The element composition of the initial materials was obtained through X-ray fluorescence (XRF) analysis, using a Bruker X-ray S8 Tiger WD spectrometer (Bruker, Billerica, MA, USA) featuring a rhodium (Rh) tube. The anode voltage was up to 60 kV, and the electric current intensity was up to 160 mA. The powder samples were processed in a helium atmosphere. The results were defined using the SPECTRA Plus QUANT EXPRESS method.

The mineral compositions (XRD) were investigated through the X-ray diffractometer DRON–7 (DINS Technologies, Glasgow, UK), with Bragg–Brentano geometry. The device applied Ni-filtered CuKα radiation and a graphite monochromator, performing with 30 kV voltage and a 20 mA emission current. The peaks corresponding to the mineral compounds were identified by using the Oxford Cryosystems Crystallographica Search-Match software, with reference to the PDF-2 database (version 3.1.0.2).

Microstructural analysis was performed with a scanning electron microscope (SEM) Hitachi S-3400N Type II, with image resolutions of secondary electrons in high vacuum. The applied acceleration voltages, for different enhancements, were 5 kV and 15 kV.

The particle size distribution of the initial materials was determined with a Cilas 1090 laser particle size analyser (Cilas, Orléans, France) operating in a dry dispersion mode, with measuring rates between 0.1 μm and 500 μm. The distribution of solid particles in the airstream was 12 wt.–15 wt.%. Compressed air (2500 mbar) was used as a dispersing phase.

The early hydration kinetics were analysed via hydration temperature analysis, employing a Pico Technology 8—channel USB TC-08 Data Logger device and a K-type thermocouple, with a measuring rate ranging between −270 °C and 1820 °C and taking 30 s.

Compressive tests were performed with the computerised press ToniTechnik 2020.0600/132/02, with a loading rate of 1.5 MPa/s (as required in EN 196-1 European Standard [17]) and a measuring rate of 0.02 s.

ANOVA and regression analyses were performed using MS Office Excel to statistically evaluate the effects of key parameters—namely the BW additive content, water-to-solid ratio, and specific surface area of the mix—on the compressive strength of the samples. ANOVA was applied to determine the significance of each factor and their interactions, while regression analysis was used to establish predictive relationships between these variables and compressive strength. This approach enabled a quantitative assessment of how mix design parameters influence mechanical performance, providing insights for optimising binder formulations.

**Figure 1:**
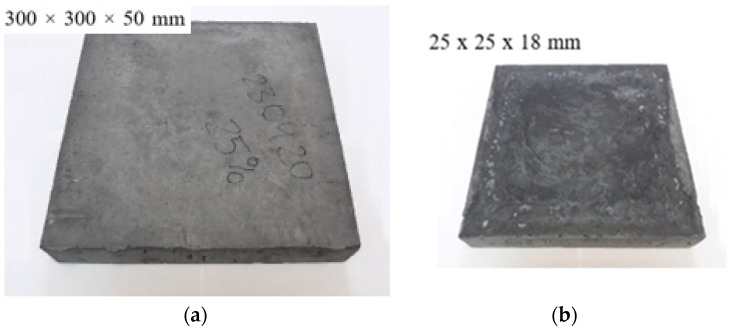
Photograph of alkali-activated slag composite samples with carbon-based BW made from wood waste for determining the following: (**a**) the thermal conductivity; (**b**) the sound isolating properties.

In accordance with standard EN 12667 [18], samples with a size of 300 × 300 × 50 mm were prepared for the determination of the thermal conductivity properties (Figure 1a). First, the samples were dried for 24 h at (110 ± 5) °C until constant weight was achieved.

A close sound-isolating frame with a speaker on one side and a microphone on the other was used for the evaluation of acoustic isolation properties of the investigated composite materials. In this case, 250 × 250 × 18 mm samples were prepared (Figure 1b) and were built into the frame to prevent sound waves from travelling from the speaker to the microphone. Visaton BG17 full-range loudspeakers (Visaton, Haan, Germany) and a Vector Research VA-1400 audio amplifier (Vector Research, Chatsworth, CA, USA) was used. The constant sound pressure level (SPL) was estimated in two ways: by measuring the total noise levels and by measuring the frequency spectrum with standard octave sound filters (31.5, 63, 125, 250, 500, 1000, 2000, 4000, and 8000 Hz).

### 2.2. Employed Materials

The main aluminosilicate precursor of the alkali-activated binder was granulated ferrous slag (Finland). The slag was milled in a laboratory ball mill. X-ray fluorescence (XRF) analysis was conducted on this initial material, indicating that calcium and silicon oxides are the dominant oxides, while small amounts of aluminium oxide and magnesium oxide were detected as well (Table 1). The additional aluminosilicate precursor was a zeolitic by-product (ZY). This by-product was obtained from an oil industry plant, and it was used without any additional pre-treatment. The zeolitic by-product was made from dominant silicon and aluminium oxides.

Phosphogypsum (PG) was introduced as the accelerator of hydration reactions in the alkali-activated slag system. PG significantly improved the geopolymerisation process and increased the mechanical properties of alkali-activated slag [19,20]. This by-product was obtained from a Lithuanian fertiliser plant. The powder PG was dried in the laboratory at 100 °C temperature. The main oxides of the PG refer to calcium and sulphur oxides, and the sum of these oxides amounts to 91.31%. In addition to the aforementioned oxides, traces of aluminium, magnesium, iron, and phosphorus oxides were also reported.

Table 1 also shows the BW composition, revealing the predominance of carbon compounds, accompanied with noticeable amounts of impurities (mainly CaO, SO_3_, SiO_2_, Fe_2_O_3_, K_2_O, Al_2_O_3_). The results of energy dispersive X-ray spectroscopy (EDX) analysis confirm the previously described XRF composition of BW, revealing that it is a carbon-based material (carbon content of 71.62%). Other chemical elements such as calcium, potassium, and aluminium are present only in trace amounts. Wood waste was the initial BW material and was produced in a Lithuanian factory by pyrolysis. The pyrolysis of wood waste lasted for 6 h at a temperature of 600 °C. After pyrolysis, BW was milled in a ball mill. A similar chemical composition of BW was determined by Prabahar et al. [13], among other studies.

**Figure 2:**
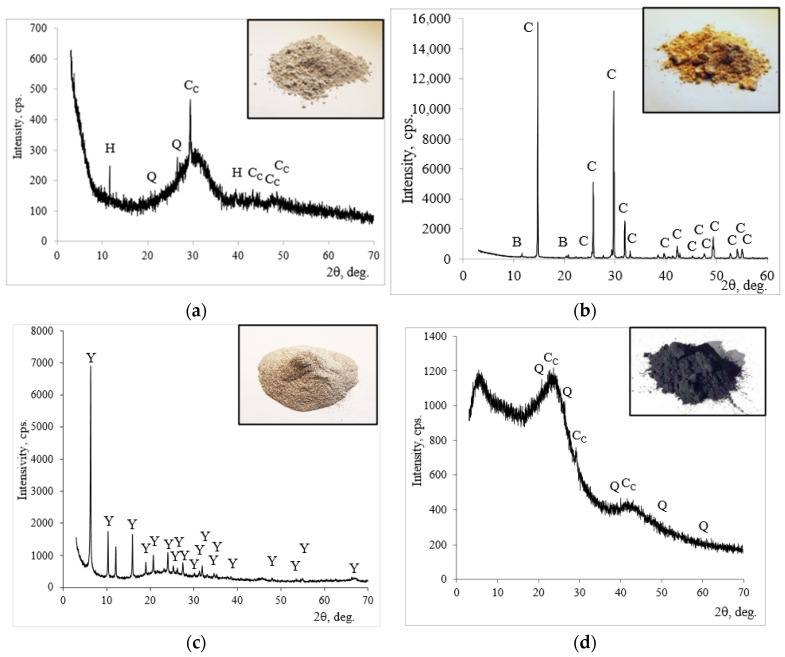
The mineral composition (X-ray diffraction patterns) of (**a**) slag; (**b**) PG; (**c**) ZY; (**d**) BW. Notes: CC—CaCO_3_ calcite (72–1937); Q—SiO_2_ quartz (83–539); H—hydrotalcite Mg_6_Al_2_CO_3_(OH)_16_∙4H_2_O (14–191); C—CaSO_4_∙0.5H_2_O basanite (33–310); B—CaPO_3_∙(OH)∙2H_2_O brushite (11–293); Y—faujasite Al_60.352_∙Si_139_∙O_371.52_∙H_5.984_ (73–2313).

The mineral composition of initial materials was determined through X-ray diffraction (XRD) analysis. Slag is a semi-amorphous semi-crystalline material (Figure 2a). Calcite, quartz, and hydrotalcite are the main minerals of slag. In addition to these crystalline compounds, amorphous SiO_2_ was identified, associated with a halo peak in the 20–35° (2θ degree) range [21]. In the PG, basanite (CaSO_4_∙0.5H_2_O) dominated and only a small amount of brushite was detected (Figure 2b). ZY consisted of faujasite (Figure 2c). BW is an almost amorphous material with low intensity peaks assigned to calcite and quartz (Figure 2d).

**Figure 3:**
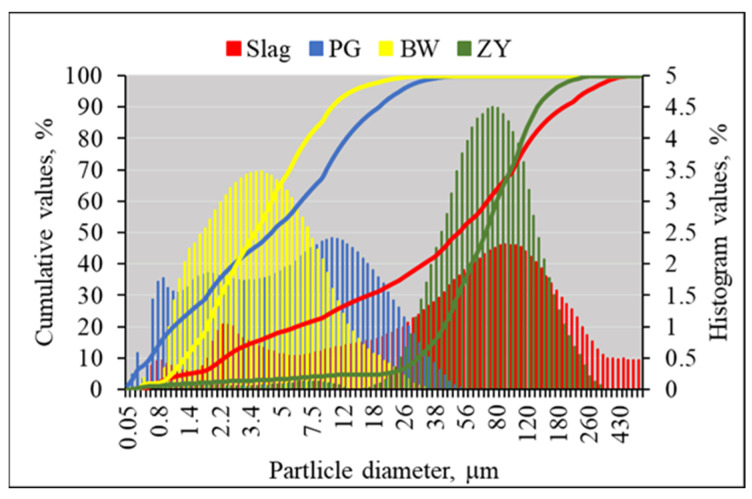
The particle size distributions: PG; slag; BW; ZY.

According to the granulometric analysis of the initial materials (Figure 3 and Table 2), BW had the finest particles among all materials: the mean diameter of particles is 5.21 µm. PG had a similar mean diameter of particles at 8.88 µm. The mean diameters of slag and ZY were almost ten times larger, having a mean diameter of 85.74 µm and 80.99 µm, respectively. The specific surface area of the initial materials was in the range of 207 m^2^/kg and 373 m^2^/kg.

**Figure 4:**
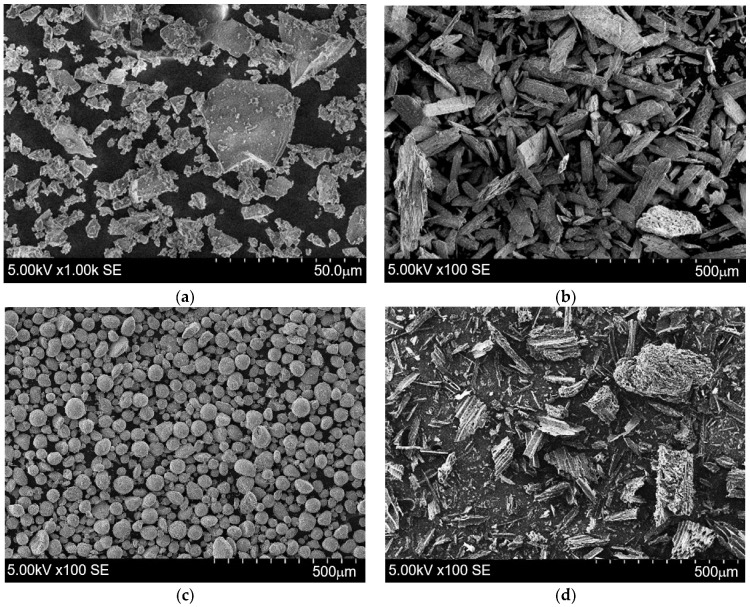
The scanning electron microscopy images of (**a**) slag; (**b**) PG; (**c**) ZY; (**d**) BW.

SEM images of the various materials are given in Figure 4. Different degrees of magnification were used because of the different particle sizes of each material. The microstructure of the slag was characterised by its irregular shape and sharp-edged particles (Figure 4a). PG mainly consisted of elongated particles (Figure 4b). The spherical shapes of particles dominate in the case of the zeolitic by-product (Figure 4c). The microstructure of BW is characterised by an irregular shape and porous conglomerates (Figure 4d). These BW insights coincide with the random shape and porous structures of BW particles, as determined by Elnour et al. [22].

A solution of sodium hydroxide (in granular form, origin—PCC Rokita, Brzeg Dolny, Poland) was prepared as an alkali activator for the powder precursor activation.

### 2.3. Preparation and Testing of the Samples

The preparation stage of the alkaline-activated material was divided into two stages. First, the dry initial materials such as slag, PG, zeolitic by-product, and BW were carefully mixed. In the second stage, the dry mixtures were blended with the solution of sodium hydroxide. The wet mass was mixed again and finally poured into the plastic moulds. At this stage, the hydration temperature test was performed. The amounts of the dry ingredients of the mixture are shown in Table 3. Twelve mixtures were prepared by changing the amount of BW, which was calculated from the sum of the precursor, as follows: slag + PG + ZY. The proportions of slag, PG, and sodium hydroxide were selected according to previous investigations by the authors [23]. The zeolitic by-product was incorporated in the mixtures due to the possibility of combining Ca(OH)_2_, which was produced from the PG and sodium sulphate. PG was used as an accelerator of the geopolymerisation process, as explained by Douglas and Brandstetr [24]. After mixing, the samples were poured into 20 × 20 × 20 mm silicone moulds and covered with polyethylene covering material to ensure good hydration. During the first day, samples hardened at room temperature. On the second day, the samples were taken to a dryer and hardened at 60 °C degrees for 24 h. A curing temperature of 60 °C combined with a curing duration of 24 h is widely recognised as optimal for achieving superior mechanical properties in alkali-activated systems [25].

**Figure 5:**
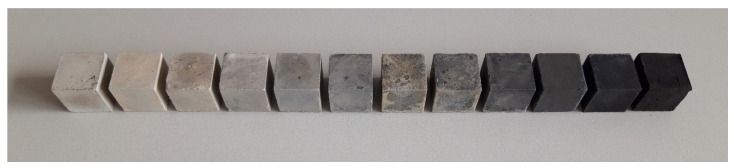
Alkali-activated slag composites with carbon-based BW made from wood waste, prepared in order to determine compressive strength (sample numbers increase from left to right).

Finally, samples were cured at room temperature for 26 days. After 7 and 28 days, samples were removed from the polythene coating, dried at 50 °C temperature for 24 h, and then the compressive strength was determined (see Figure 5). It should be mentioned that efflorescence was detected. Moreover, XRD analysis of the specimens exhibiting the most relevant results was performed.

## 3. Results and Discussion

The results of the various tests are further described in this section, including the following: compressive strength, hydration dynamics, and XRD and SEM characterisation of representative samples. The insights of ANOVA and regression analysis to explain the compressive strength dependence on three factors (amount of BW additive, specific surface of precursors, and amount of mixing water) are also provided and discussed. Moreover, the thermal and acoustic insulation properties of the specimens are analysed.

### 3.1. Compressive Strength Properties of Composite Samples

**Figure 6:**
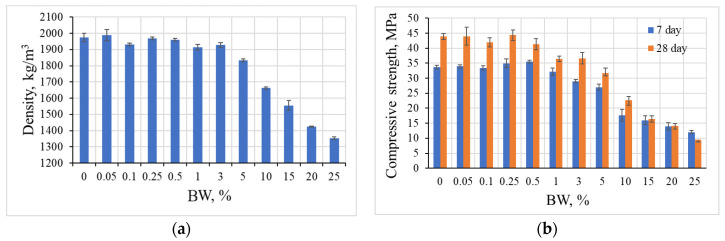
Dependences of density (**a**) and compressive strength (**b**) on the amount of BW additive in alkali-activated slag composite samples.

The results of the density and compressive strength of the samples are provided in Figure 6, which reveals that the density of alkali-activated slag is strongly correlated with the amount of BW. The samples exhibited two density tendencies, depending on the amount of additive: up to 0.5% of BW, the values of density were similar to the reference samples, but with an amount of BW larger than 1%, the density decreased (see Figure 6a); additionally, with an amount of BW up to 3% the decrease is slight, but with 5% and higher the density reduction becomes significantly pronounced. The compressive strength results showed similar tendencies (see Figure 6b): with an increase in BW up to 0.5%, the 7-day compressive strength values gradually increased from 33.7 MPa to 35.6 MPa. The opposite trend was noted in that case when BW was added in larger amounts from 1% to 25%. In this case, the compressive strength gradually decreased. After 28 days the highest compressive strength (44.4 MPa) was determined for the samples with 0.25% BW; this was slightly higher when compared to the reference samples without BW. By comparing the results after 7 and 28 days of hydration, higher compressive strength was reached after a longer hydration period, as expected.

The development of compressive strength after 7 days could be explained by the effect of crystallisation centres. Presumably, as Asadi et al. [26] explains, smaller amounts (0.05–0.5%) of BW incorporated into alkali-activated slag systems acted as crystallisation centres which help to form hydration products. BW being a micro aggregate slightly improved compressive strength after 28 days (Ferro [27]). In this case, the micro cracking of composite matrixes was reduced due to the homogeneous distribution. When a higher amount of BW (1–25%) was incorporated, the BW distribution was non-homogeneous and this led to a reduction in the mechanical properties, as has been previously explained by Sundarakannan et al. [28].

### 3.2. Single-Factor ANOVA Evaluation Method for the Compressive Strength of the Samples

The single-factor ANOVA tool performs a statistical comparison of two or more sample data sets to determine whether their means differ significantly. It tests the null hypothesis that all samples originate from the same probability distribution against the alternative hypothesis that at least one sample differs. The method calculates an F-statistic, which is the ratio of variance between groups to variance within groups. A larger F-value indicates that the differences between group means are unlikely to be due to random variation, suggesting a real effect. A large F-value than F-crit indicates real differences between the factors under investigation. When the calculated F-value is less than F-crit, this indicates that the factors under investigation are independent.

Counts represent the sample number of observations in each group, while average denotes the mean compressive strength. Excel displays averages with up to six decimal places to maintain precision during F-statistic computation; rounding is applied only to the final F-value (typically to two decimal places) for clarity in reporting.

The impact of the BW additive, specific surface area of precursors, and the amount of water on the compressive strength of alkali-activated slag composites were evaluated. These three factors were considered as parameters. A feasible range of parameters was determined through twelve mixes and the blend design was optimised with reference to compressive strength.

ANOVA analysis of variance between the BW additive content and compressive strength revealed that the calculated *F*-test value (31.52) is many times higher than the *F*-crit value (Table 4). This shows that the amount of BW additive in the samples significantly affects their compressive strength. A similar tendency is noted when analysing the ANOVA analysis of variance between the specific surface area of the mixture and the compressive strength of the samples (Table 5). Only in this case does the higher *F*-test value prove that the specific surface area of the mixtures has a stronger influence on the samples than the amount of BW additive.

After performing the ANOVA analysis of variance between water content and compressive strength, it was found that the calculated *F*-test value (31.52) is lower than the *F*-crit value (Table 6). This proves that the variation in the amount of water used had no significant effect on the compressive strength of the samples when compared with two other factors such as the BW additive content and specific surface area of the mixtures.

**Figure 7:**
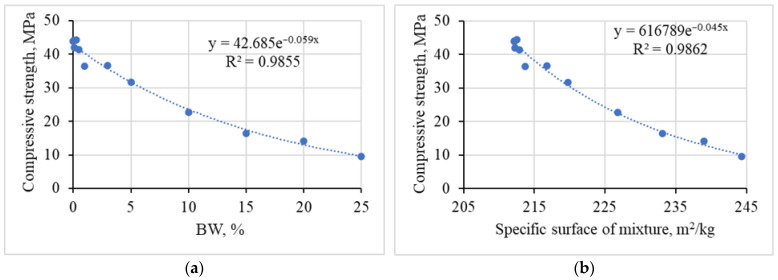
The dependence of the compressive strength of the samples on significant factors: regression analysis of BW amount (**a**) and specific surface area of mixtures (**b**).

After carrying out a regression analysis of the dependence of the compressive strength of the samples on significant factors, it was found that in both cases the results of the tests are best described by the exponential equation (Figure 7). The calculated *R*^2^ value of the coefficient of determination (>0.98) shows that the variation in the compressive strength of the samples can be explained by the variation in both BW% and specific surface area of the mixture values.

The calculated *R*^2^ value of the coefficient of determination (≥0.98) shows that the variation in the compressive strength of the samples can be explained by the variation in both BW and the specific surface areas of the mixture values. Similar observations are reported in the work of Prabahar et al. [13]. By using an optimal amount of BW powder, the strength enhancement at an early period increased.

### 3.3. Mineral Composition and Hydration Temperature of Alkali-Activated Slag Systems with BW

**Figure 8:**
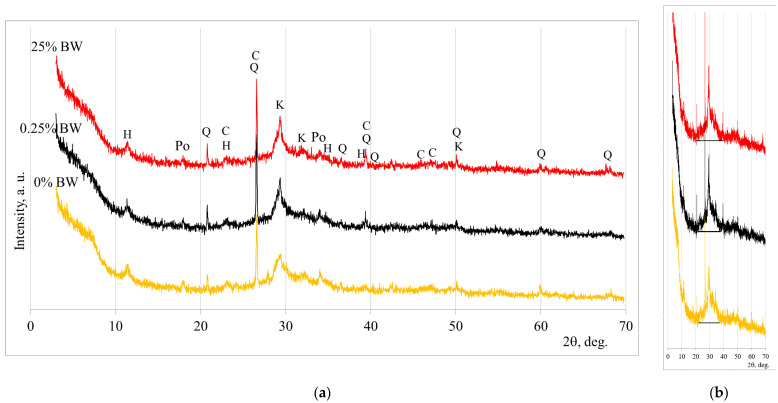
XRD curves of alkali-activated slag with (0.25%, 25%) of BW and without BW (0%). The curves for crystalline compounds identification (**a**) and the same curves for comparing the amorphous hump (**b**). Notes: Q—(83–539) quartz SiO_2_, K—(33–306) calcium silicate hydrate Ca_1.5_SiO_3.5_∙H_2_O, C—(72–1651) calcite CaCO_3_, H—(14–191) hydrotalcite Mg_6_Al_2_CO_3_(OH)_16_∙4H_2_O, Ca—(70–845) calcium aluminium silicate hydrate Ca_5.57_Al_12.3_Si_12_O_49.2_H_2.34_, Po—(70–845), Po—(1–1079) portlandite Ca(OH)_2_.

Figure 8 presents the XRD mineral composition of representative samples after 28 days of hydration. Three samples were selected: the control sample (without BW), the sample exhibiting the highest compressive strength values (0.25% of BW), and the sample with maximum amount of BW (25%), respectively. In fact, similar mineral composition was detected in all samples. It can be noticed that crystalline compounds of calcium silicate hydrate, calcite, calcium aluminium silicate hydrate, and portlandite were formed through the hydration process (Figure 8a). Meanwhile, calcite, quartz, and hydrotalcite, which were found in the slag and BW as initial materials, remained unreacted after the hydration process.

Thus, the studied XRD curves have the same peaks, but the intensity of peaks which are attributed to calcium silicate hydrate are different. It was noted that the incorporation of BW led to an increase in the intensity of the main peak (at 27.5) of calcium silicate hydrate (a similar tendency is explained by Kim et al. [29]). In addition to crystalline compounds, the hydration products also contain amorphous ones. The halo peak (amorphous hump) around 25° to 35° is detected for all samples and its area is similar in all cases, as indicated in Figure 8b; this was also detected in other studies [14,30].

**Figure 9:**
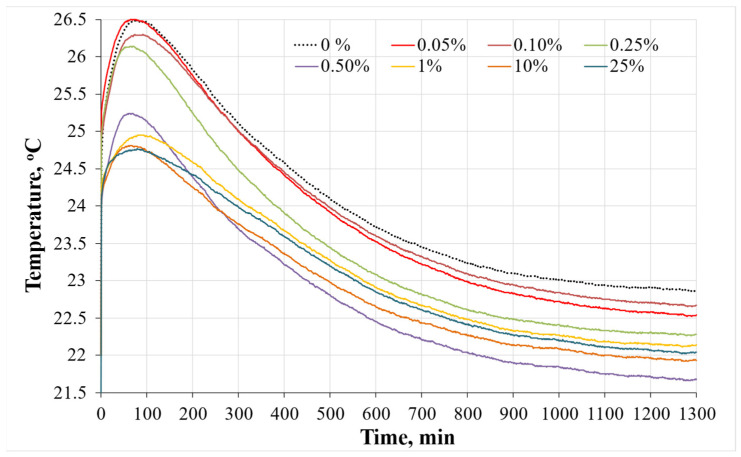
The curves of hydration temperature of composite samples without BW (0%) and with (0.05–25%) BW.

Figure 9 presents the change in temperature during hydration. The highest temperature (26.6 °C) was reached during hydration of the samples with the lowest amount of BW (0.05%). This temperature was similar to the reference sample without BW (26.5 °C). With increasing BW content, the hydration temperature peaks gradually decreased. This decrease could be explained with reference to the dilution effect. BW is not a reactive material and acted as an inert filler, which modified the hydration mechanism, as explained by Chen et al. [31]. For this reason, in the alkali-activated slag system, there is a lower amount of precursors from which the hydration products are formed. The time when the maximum hydration temperature was reached was evaluated as well. It was determined that this time became shorter with increasing BW content in the alkali-activated slag systems. A similar finding was found in another study by Egodagamage et al. [8] where it was reported that the BW made from rice husk speeds up the hydration process. In another study by Gupta et al. [32], the acceleration of hydration was also determined. In this case, BW from mixed wood saw dust acted as inert micro-filler, which was responsible for a slightly accelerated hydration process.

### 3.4. Morphological Characterisation

**Figure 10:**
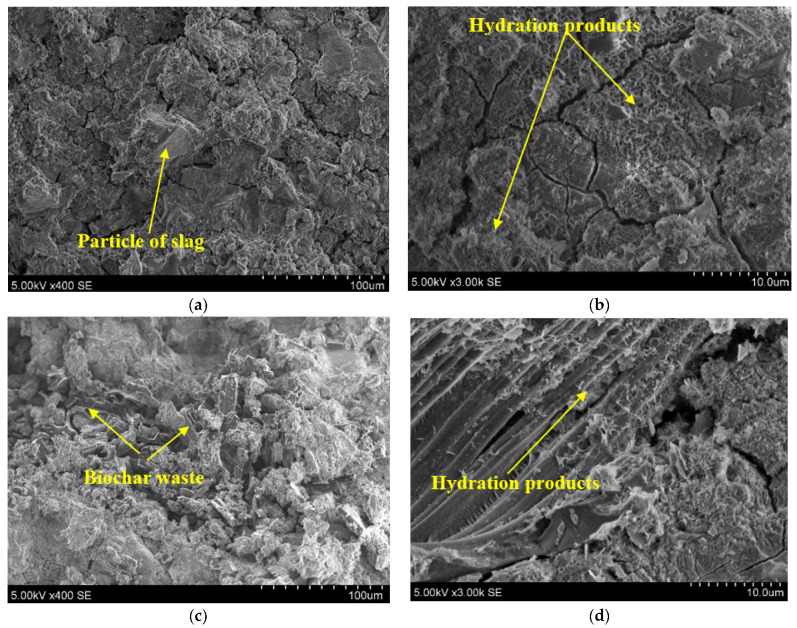
Scanning electron microscope (SEM) images of alkali-activated slag and BW composites at different magnifications. Notes: (**a**,**b**) are composite without BW; (**c**,**d**) are composite with 0.25% BW.

The microstructure of the binding systems is closely related to their main mechanical properties and for that reason it is important to investigate it in detail. Figure 10 shows the SEM microstructure of two representative alkali-activated slag samples. The first one (see Figure 10a,b) was a reference sample without BW, presented with different magnifications (×400 and ×3000, respectively). A slag particle that does not react in an alkaline environment is visible in Figure 10a. Hydration products such as calcium silicate hydrate (CSH), as a crystalline compound, and CASH, a gel with an amorphous compound, were detected at a larger magnification (Figure 10b). Other researchers, such as Jia et al. [33], stated that CASH and CSH are the main hydration products of alkali-activated slag. In the analysed sample, micro-cracks were also observed. They are likely formed due to drying shrinkage in the alkali-activated slag paste, as also described by Yang et al. [34].

In the sample with BW additions, hydration products in the pores and channels of the BW could be detected, as noted in Figure 10c,d. For this reason, BW particles had a good contact zone with the matrix of the alkali-activated slag, since hydration products crystallised inside the BW. Sirico et al. [35] performed a SEM analysis and reported that there is no interphase separation between BW and concrete. Moreover, a much lower number of micro-cracks were detected in the composite with BW. During mixing, BW absorbs water and, after some time, releases water during the hydration process. Therefore, BW acts as a water reservoir. During hydration the additional amount of water and space for hydration products is accrued in the BW, as indicated by Dixit et al. [8]. Similar findings were published by Liu et al. [36], who discovered that mortar with 1% bamboo BW had a higher cracking resistance when comparing with a reference mortar.

### 3.5. The Thermal Conductivity and Acoustic Isolation Properties

**Figure 11:**
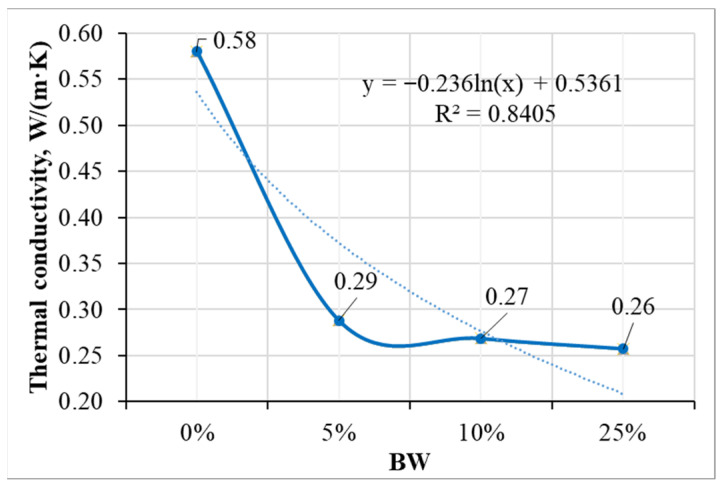
The influence of BW amount in the composite on the thermal conductivity of samples.

The BW incorporated in alkali-activated slag composite had significant influence on the thermal conductivity. A strong linear relationship between density and thermal conductivity was obtained (see Figure 11). By increasing the amount of BW, thermal conductivity decreased. The addition of 5% BW reduced the thermal conductivity values by almost twice the original value, from 0.58 W/(m·K) to 0.29 W/(m·K). This reduction could be related to the lower density and higher porosity of composites with BW. Furthermore, with higher amount of BW (10% and 25%), the thermal conductivity did not change significantly: it slightly decreased to 0.27 W/(m·K) and 0.26 W/(m·K) with 10% and 25% of BW, respectively. Boumaaza et al. [37] published similar thermal conductivity values of the mortar with palm tree BW, where thermal conductivity was in the range of 0.33–0.45 W/(m·K) when using 1 and 3% BW, respectively.

Because excessive noise negatively affects humans, it is important to protect buildings from noise pollution. Improved sound control is achieved through acoustic insulation; the insulation creates a sound barrier, blocking out unwanted sounds. In this study, samples of alkali-activated slag with BW were used for noise pollution protection.

**Figure 12:**
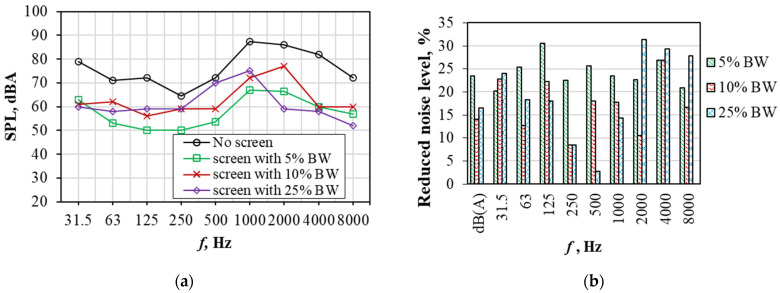
Dependence of the sound pressure level (SPL) (**a**) and the reduction noise level (**b**) on the frequency spectrum.

The reduction in noise level (Figure 12a) and SPL (Figure 12b) was determined by using different types of samples: alkali-activated slag with 5%, 10%, and 25% of BW. It was determined that SPL without the screen showed higher values when compared to SPL results with composites screens. The lowest values of SPL were obtained for the sample with 5% BW and reached 50 dBA in the range of 63–500 Hz. However, humans are more sensitive to high frequency diapason (from 1500 Hz to 8000 Hz). So, in this diapason, the lowest SPL was determined for the sample with the highest BW content (25%) and the reduction in noise was about 30% when compared with the noise level obtained without the sound barrier.

## 4. Conclusions

This paper is a study on the valorisation of biochar waste (BW) in building materials, investigating its performance as an additive or a filler in an alkali-activated concrete matrix. The provided insights and recommendations are valuable due to the large volume of such waste and the need to manage it at different efficiencies, which may compete with its current use or disposal. The potential of the suggested material was evaluated though three main properties: density, thermal and acoustic insulation, and alkaline activation.

- The compressive strength of alkali-activated slag with smaller amounts of BW (0.05–0.5%) slightly increased due to a high resistance to crack propagation, achieving the highest 28-day strength value of 44 MPa with 0.5% of BW. The homogeneous distribution BW’s porous nature led to slight improvements in compressive strength. By using a larger amount of BW, the mechanical properties decreased due to the non-homogeneous distribution of BW in the composite.
- Using mathematical analysis methods, it was proven that the compressive strength of the samples under the investigated conditions is mainly influenced by the specific surface area of the mixture components and the amount of BW additive. It was established that the variation in the compressive strength values of the samples with specific surface of mixture and BW is best described by the exponential equation.
- The mineral composition of samples was affected by the amount of BW. More intensive peaks of calcium silicate hydrate were determined for the samples with BW, and the powder of BW acted as nucleation sites for calcium silicate hydrate. The hydration temperatures slightly decreased due to the BW’s action as an inert micro-filler.
- The particles of BW show a very good compatibility with the alkali-activated slag matrix. No interphase separation between BW and concrete occur, as detected through SEM analyses.
- The thermal conductivity of alkali-activated slag composite with BW was almost two times lower than the thermal conductivity of the reference samples without BW. The addition of 5%, 10%, and 25% BW decreased the thermal conductivity values from 0.58 W/(m·K) to 0.29 W/(m·K), 0.27 W/(m·K),and 0.26 W/(m·K) with 5%, 10%, and 25% of BW, respectively. The acoustic insulation effect of the alkali-activated slag composite containing 25% BW was most pronounced, achieving the lowest SPL value of 52 dB in the high-frequency range, compared to composites with 5% and 10% BW, which recorded SPL values of 57 dB and 60 dB, respectively.

The addition of a low-cost and sustainable carbon-based additive (0.05–0.50% BW) and filler (0.5–25% BW) to the alkali-activated slag matrix can create new sustainable materials that have low thermal conductivity and possess an adequate acoustic insulation effect.

## Figures and Tables

**Table 1:** Oxide composition of the initial materials (XRF analysis), wt.%.

Initial Materials	SiO_2_	CaO	Al_2_O_3_	Fe_2_O_3_	MgO	K_2_O	P_2_O_5_	SO_3_	BaO	SrO	TiO_2_	C	Other
Slag	37.4	45.3	6.54	0.79	5.99	0.67	0.07	1.85	0.07	0.07	0.29		0.96
PG	0.34	39.16	0.08	0.04	0.2		1.63	52.15					6.4
ZY	38.55	0.69	49.57	1.06	0.66		0.08	0.07			3.55		5.77
BW	3.8	4.86	2.05	3.7	1.35	2.12	3.06	4.07	0.59	0.85	0.15	71.62	1.78

**Table 2:** The particle size distribution parameters of the initial materials.

Parameters	Initial Materials			
	PG	Slag	ZY	BW
Diameter sizes, µm:				
Amount of particles, 10%	0.74	2.55	31.46	1.41
Amount of particles, 50%	4.63	54.85	69.67	3.76
Amount of particles, 90%	19.98	202.89	140.04	10.81
Mean, μm	8.88	85.74	80.99	5.21
Specific surface, m^2^/kg	318	207	248	373

**Table 3:** The composition of the mixtures for the initial materials, wt.%.

Sample No.	Slag	PG	ZY	BW *	NaOH *	H_2_O *
1	92.38	2.86	4.76	0	9.71	25.40
2	92.38	2.86	4.76	0.05	9.71	25.40
3	92.38	2.86	4.76	0.1	9.71	25.40
4	92.38	2.86	4.76	0.25	9.71	25.40
5	92.38	2.86	4.76	0.5	9.71	26.98
6	92.38	2.86	4.76	1	9.71	26.98
7	92.38	2.86	4.76	3	9.71	28.57
8	92.38	2.86	4.76	5	9.71	33.33
9	92.38	2.86	4.76	10	9.71	39.68
10	92.38	2.86	4.76	15	9.71	46.03
11	92.38	2.86	4.76	20	9.71	53.97
12	92.38	2.86	4.76	25	9.71	63.49

* Calculated from the amount of precursor: the mixtures of slag, PG, and ZY.

**Table 4:** ANOVA single factor for BW additives content and compressive strength.

**SUMMARY**						
**Groups**	**Count**	**Sum**	**Average**	**Variance**		
BW, %	12	79.9	6.658333	77.57492		
Compressive strength at 28 days	12	383.2174	31.9348	165.6648		
**ANOVA**						
**Source of Variation**	**SS**	**df**	**MS**	**F**	***p*** **-value**	***F*-crit**
Between Groups	3833.394	1	3833.394	31.52	1.21·10^−5^	4.30
Within Groups	2675.636	22	121.620			
Total	6509.031	23				

**Table 5:** ANOVA single factor for specific surface area of mixtures and compressive strength.

**SUMMARY**						
**Groups**	**Count**	**Sum**	**Average**	**Variance**		
Specific surface area	12	2655.492	221.291	134.946		
Compressive strength at 28 days	12	383.2174	31.93479	165.6648		
**ANOVA**						
**Source of Variation**	**SS**	**df**	**MS**	**F**	***p*** **-value**	***F*-crit**
Between Groups	215,134.6	1	215,134.6	1431	1.62·10^−21^	4.30
Within Groups	3306.719	22	150.3054			
Total	218,441.3	23				

**Table 6:** ANOVA single factor for water and compressive strength.

**SUMMARY**						
**Groups**	**Count**	**Sum**	**Average**	**Variance**		
H_2_O	12	2655.492	221.291	134.946		
Compressive strength at 28 days	12	383.2174	31.93479	165.6648		
**ANOVA**						
**Source of Variation**	**SS**	**df**	**MS**	**F**	***p*** **-value**	***F*-crit**
Between Groups	58.32087	1	58.32087	0.35	0.5608	4.30
Within Groups	3678.798	22	167.2181			
Total	3737.119	23				

## Data Availability

The original contributions presented in this study are included in the article. Further inquiries can be directed to the corresponding author.

## Abbreviations

The following abbreviations are used in this manuscript:

BW	Biochar waste
SEM	Scanning electron microscopy
XRD	X-ray diffraction
XRF	X-ray fluorescence
SPL	Sound pressure level
PG	Phosphogypsum
ZY	Zeolitic by-product
